# Gemcitabine-Based Chemoradiotherapy Enhanced by a PARP Inhibitor in Pancreatic Cancer Cell Lines

**DOI:** 10.3390/ijms22136825

**Published:** 2021-06-25

**Authors:** Waisse Waissi, Jean-Christophe Amé, Carole Mura, Georges Noël, Hélène Burckel

**Affiliations:** 1 Department of Radiation Oncology, Centre Leon Bérard, 69008 Lyon, France; waisse.waissi@lyon.unicancer.fr; 2 Poly(ADP-ribosyl)ation and Genome Integrity, Laboratoire d’Excellence Medalis, UMR7242, Centre Nationale de la Recherche Scientifique/Université de Strasbourg, Institut de Recherche de l’Ecole de Biotechnologie de Strasbourg, 300 bld. S. Brant, CS10413, 67412 Illkirch, France; ame@unistra.fr; 3 Institut de Cancérologie Strasbourg Europe (ICANS), Strasbourg University, UNICANCER, Paul Strauss Comprehensive Cancer Center, Radiobiology Laboratory, 67000 Strasbourg, France; c.mura@icans.eu; 4 Institut de Cancérologie Strasbourg Europe (ICANS), UNICANCER, Paul Strauss Comprehensive Cancer Center, Department of Radiation Oncology, 17 Rue Albert Calmette, 67200 Strasbourg, France; g.noel@icans.eu; 5 Strasbourg University, CNRS, IPHC, UMR 7178, 67200 Strasbourg, France

**Keywords:** pancreatic adenocarcinoma, PARP inhibitor, Gemcitabine, irradiation, radiosensitizer

## Abstract

Pancreatic ductal adenocarcinoma is a devastating disease with a 5-year overall survival of 9% for all stages. Gemcitabine-based chemoradiotherapy for locally advanced pancreatic cancer is highly toxic. We conducted an in vitro study to determine whether poly(ADP-ribose) polymerase-1 inhibition radiosensitized gemcitabine-based chemotherapy. Human pancreatic cancer cell lines, MIA PaCa-2, AsPC-1, BxPC-3 and PANC-1 were treated with gemcitabine (10 nM) and/or olaparib (1 µM). Low-LET gamma single dose of 2, 5 and 10 Gy radiations were carried out. Clonogenic assay, PAR immunoblotting, cell cycle distribution, γH2Ax, necrotic and autophagic cell death quantifications were performed. Treatment with olaparib alone was not cytotoxic, but highly radiosensitized cell lines, particularly at high dose per fraction A non-cytotoxic concentration of gemcitabine radiosensitized cells, but less than olaparib. Interestingly, olaparib significantly enhanced gemcitabine-based radiosensitization in PDAC cell lines with synergistic effect in BxPC-3 cell line. All cell lines were radiosensitized by the combination of gemcitabine and olaparib, through an increase of unrepaired double-strand, a G2 phase block and cell death. Radiosensitization was increased with high dose of radiation. The combination of olaparib with gemcitabine-based chemoradiotherapy could lead to an enhancement of local control in vivo and an improvement in disease-free survival.

## 1. Introduction

Pancreatic ductal adenocarcinoma (PDAC) has a very poor prognosis with a 5-year overall survival of approximately 9% [1]. Surgery remains the only means of cure. However, at the time of diagnosis, up to 30% of patients present with unresectable locally advanced pancreatic cancer. In a subset of these patients, failing to control the primary tumor may result in complications that can lead to death. Therefore, making unresectable tumors resectable may improve outcomes.

As gemcitabine has been shown to both be effective and to enhance radiosensitivity on PDAC cells, chemoradiotherapy (CRT) with gemcitabine is one of the current effective option for treating non-metastatic unresectable pancreatic cancer [2]. However, gemcitabine-based CRT has a high of rate toxicity, leading to a reduction of gemcitabine dose or chemotherapy discontinuation. Using modern techniques of radiotherapy such as intensity-modulated radiotherapy (IMRT) or stereotactic body radiotherapy (SBRT) may simultaneously enhance dose to tumor and decrease dose to organ-at-risk, leading to a reduction of gemcitabine-radiation related toxicity. Besides targeted therapies that could be radiosensitizers and/or chemopotentiating agents may enhance this synergy.

Recently, DNA repair has become a major topic of investigation for the treatment of cancer. The key determinant of cellular radiosensitivity is the capacity of cells to repair highly lethal DNA double-strand breaks (DSBs). Therefore, targeting proteins implicated in the response to ionizing radiation-induced DNA damage, such as poly(ADP-ribose) polymerase-1 (PARP-1), may be an appropriate strategy. Indeed, PARP-1 is a major DNA damage sensor allowing the recruitment of DNA repair proteins involved in DNA single- and double-strand breaks repair [3]. At the outset of PARP inhibitors (PARPi) development, targeting BRCA1/2 mutant tumors has been the main approach and is known as the concept of synthetic lethality. Ongoing phase III trials are testing olaparib in different tumor types with BRCA1/2 mutations such as breast cancer (clinical trial.gov NCT02032823, NCT02000622), ovarian cancer (NCT01844986, NCT01874363) and pancreatic cancer (NCT02184195). More recently, the use of PARPi has been investigated on BRCA1/2 wild-type tumors in combination with radiotherapy [3]. Olaparib has been one of the most studied PARPi, which potentiated the effect of radiotherapy in different cell lines and tumor xenograft models [4]. However, association of olaparib, gemcitabine and irradiation has never been assessed on BRCA wild type pancreatic cancer cells yet. The main purpose of this study is to evaluate the in vitro antitumor efficacy of the association of olaparib (PARPi) and gemcitabine, combined with different radiation doses, on four pancreatic cancer cell lines.

## 2. Results

### 2.1. Cytotoxicity of Gemcitabine or Olaparib on PDAC Cell Lines

**Figure 1:**
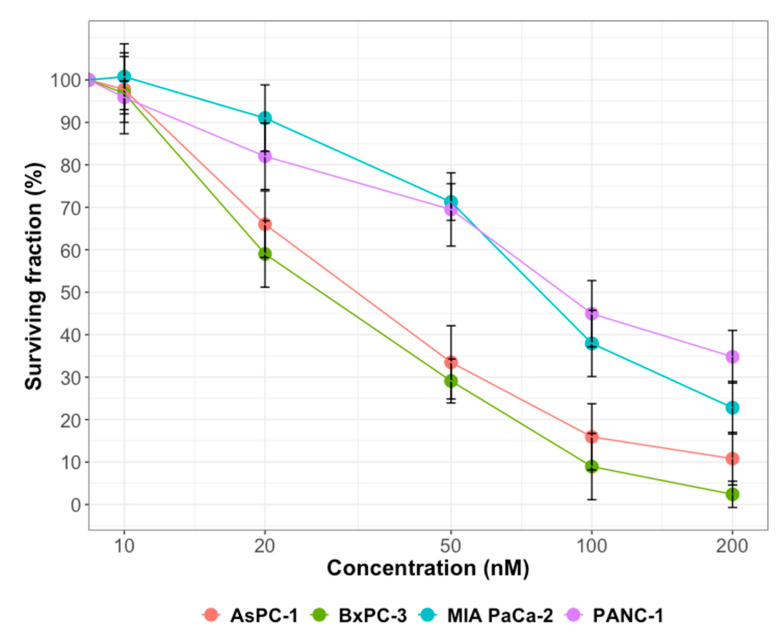
Cell survival of PDAC cell lines, AsPC-1, BxPC-3, MIA PaCa-2 and PANC-1, after gemcitabine treatments. Cells were treated with 10 to 200 nM gemcitabine for 24 h and cell survival was determined using clonogenic assay. Experiments were done in triplicate and represent mean with standard error.

To assess long term differential inhibition with gemcitabine, we conducted a clonogenic survival assay and showed that 10 nM of gemcitabine was not cytotoxic for all PDAC cell lines (Figure 1). However, beyond this concentration, cell lines were differentially sensitive to gemcitabine. Indeed, at 200 µM gemcitabine, surviving fractions were 35%, 23%, 11% and 2% for PANC-1, MIA PaCa-2, AsPC-1 and BxPC-3 cells, respectively. Finally, we confirmed that olaparib, at a concentration of 1 µM, did not significantly decrease long-term surviving fraction on the four pancreatic cell lines (Table 1). Taking together, these data displayed that olaparib at a concentration of 1 µM and gemcitabine at 10 nM were not toxic for PDAC cell lines.

### 2.2. Effect of Olaparib on PARylation

**Figure 2:**
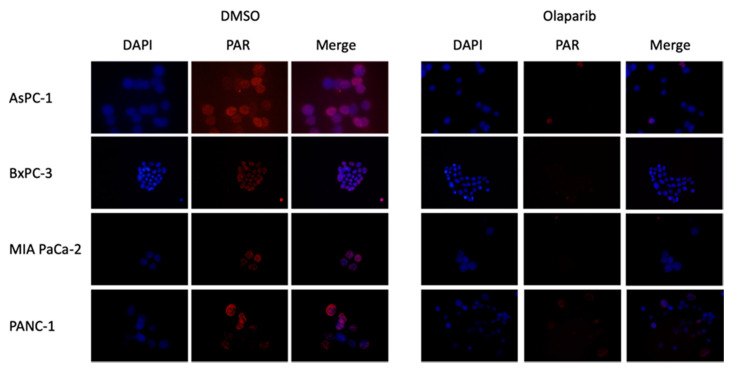
Immunodetection of poly ADP-ribose (PAR) in AsPC-1, BxPC-3, MIA PaCa-2 and PANC-1 cell lines either untreated (DMSO) or treated with olaparib (1 µM). DNA is counterstained with DAPI. Magnification 40×.

Cells were treated with H_2_O_2_ to induce replicative stress. After pretreatment with olaparib at 1 µM, PARP-1 activity was significantly decreased in the four PDAC cell lines (Figure 2). As Olaparib at 1 µM was not cytotoxic for PDAC cell lines, but significantly decreased poly ADP-ribosylation (PARylation) level, this concentration was selected for subsequent experiments.

### 2.3. Radiosensitization of Gemcitabine and/or Olaparib on PDAC Cell Lines

**Figure 3:**
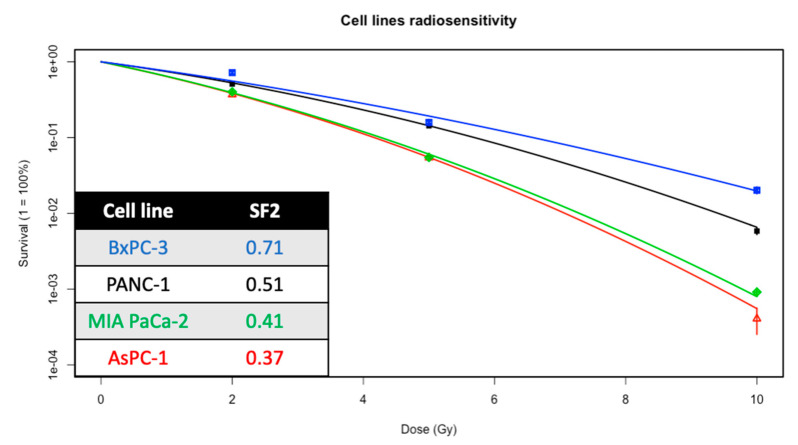
Clonogenic survival curves of PANC-1, AsPC-1, MIA PaCa-2 and BxPC-3 cell lines treated with increasing dose of irradiation (2, 5 and 10 Gy). Experiments were done in triplicate (n = 4) and represent mean with standard error.

A broad range of intrinsic sensitivity was identified across PDAC cell lines as presented in Figure 3. Among the four cell lines, BxPC-3 is the most radioresistant (surviving fraction at 2 Gy, SF2 = 0.71) and AsPC-1, the most radiosensitive (SF2 = 0.37). MIA PaCa-2 cell line has a more or less similar radiosensitivity as AsPC-1 cell line.

**Figure 4:**
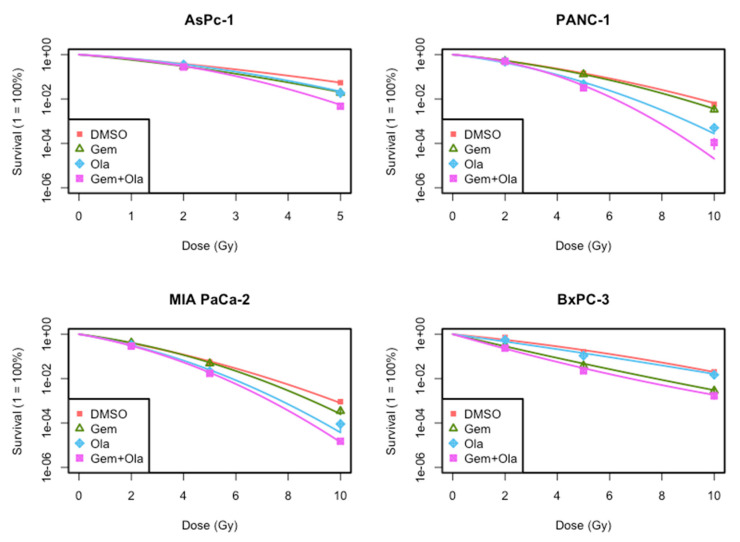
Clonogenic survival curves presenting surviving fractions of PANC-1, AsPC-1, MIA PaCa-2 and BxPC-3 cell lines treated with gemcitabine (Gem, 10 nM), olaparib (Ola, 1 µM) or the association of gemcitabine and olaparib (Gem + Ola).

To investigate intrinsic radiosensitivity and validate that a one-hour pre-treatment with olaparib and a 24 h pre-treatment with gemcitabine enhanced radiosensitivity in PDAC cell lines, clonogenic assays were performed (Figure 4). Olaparib highly radiosensitized MIA PaCa-2, AsPC-1 and PANC-1 cell lines (Table 2), particularly at high dose per fraction (10 Gy). In contrast, olaparib did not radiosensitize BxPC-3 cell line (Table 2).

Simultaneously, a non-cytotoxic concentration of gemcitabine radiosensitized MIA PaCa-2 and PANC-1 cells, but substantially less than olaparib (SER = 1.24 ± 0.001 for MIA PaCa-2 and SER = 1.38 ± 0.01 for PANC-1, Table 3). In contrast, BxPC-3 cell line was highly radiosensitized by gemcitabine (SER = 1.75 ± 0.005). Finally, AsPC-1 was radiosensitized by either gemcitabine or olaparib at the same level (Table 2). Combination of gemcitabine and olaparib enhanced gemcitabine-based chemoradiotherapy in all cell lines and showed a significant synergistic effect in BxPC-3 cell line.

### 2.4. Effect of the Treatments on Cell Cycle

**Figure 5:**
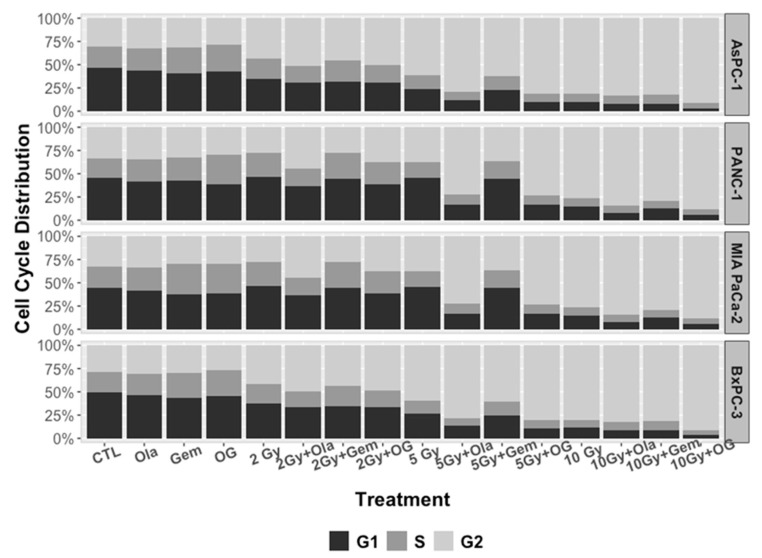
Cell cycle distribution of AsPC-1, PANC-1, MIA PaCa-2 and BxPC-3 cell lines, after irradiation (2, 5 and 10 Gy) and treatment with olaparib (1 µM) and/or gemcitabine (10 nM).

As expected, analysis of cell cycle distribution using flow cytometry (Figure 5) revealed that irradiation induced a G2/M phase arrest 24 h after irradiation and the percentage of cells in G2/M phase expanded with increasing radiation doses (2 to 10 Gy). Moreover, the association of olaparib and irradiation enhanced cell cycle arrest in G2/M phase, 24 h after irradiation. Concurrently, gemcitabine alone increased cell cycle arrest in S-phase (Table 3). Cells co-treated with gemcitabine and olaparib with or without irradiation, did not significantly modify cell cycle distribution, compared to olaparib alone. Taken together, these results showed that olaparib and irradiation had a combined effect on cell cycle arrest in G2-phase, whereas gemcitabine blocked cells in early S-phase, independently of olaparib and/or irradiation treatment.

### 2.5. Effect of the Treatments on Persistent γ-H2AX Foci

**Figure 6:**
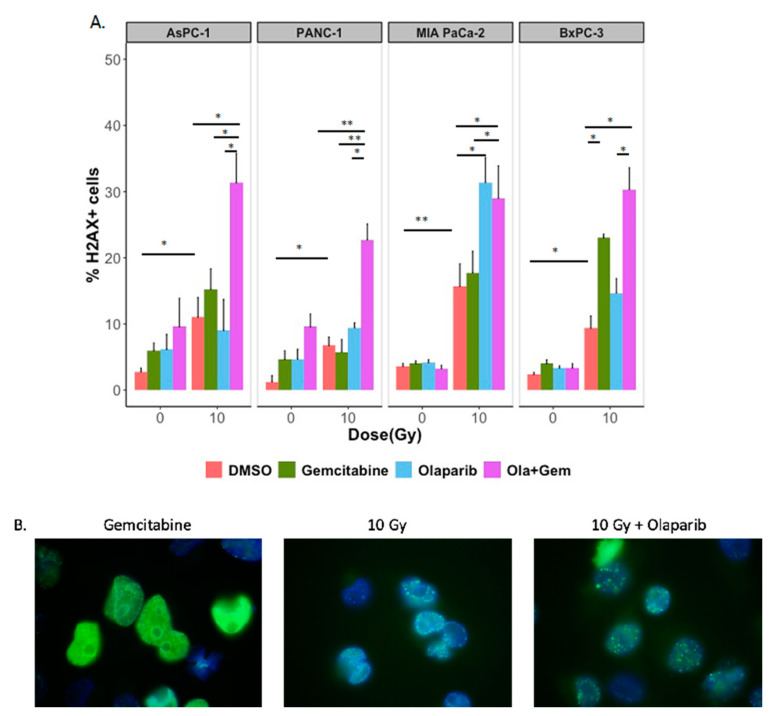
(**A**) Effect of irradiation (10 Gy), olaparib (1 µM) and gemcitabine (10 nM) on the amount of γ-H2AX positive cells, 24 h after treatment on AsPC-1, PANC-1, MIA PaCa-2 and BxPC-3 cell lines. Cells were labelled 24 h after treatment with anti- γ-H2AX antibody and analyzed for γ-H2AX positivity and DNA content using flow cytometry. Data shown are the mean percentage of γ-H2AX positive cells from 4 independent experiments. Statistical significance is indicated: *p* < 0.05 (*), *p* < 0.01 (**). (**B**) Typical patterns of γ-H2AX staining observed after treatment with gemcitabine, olaparib and 10 Gy irradiation after immunofluorescence analysis. Magnification 100×.

We recorded foci corresponding to the persistent activation of γ-H2AX in control and treated cells, 24 h after the exposure to gemcitabine, olaparib or the combination of both treatments with (10 Gy) or without irradiation (Figure 6A). As expected, the number of persistent γ-H2AX foci after irradiation increased according to the dose and after pre-treatment with olaparib, with a strong rise for MIA PaCa-2. We found that the addition of olaparib with gemcitabine did not consistently increase the number of residual γ-H2AX foci after treatment on all four PDAC cell lines.

24 h-residual double-strand breaks are generally irreparable and may induce cell death. On the side, qualitative differences were noticed in staining patterns of γ-H2AX. Indeed, cells treated with olaparib formed discrete punctuated and well-defined foci, compared with cells treated with gemcitabine that displayed a diffuse, pan-nuclear γ-H2AX staining pattern (Figure 6B).

### 2.6. Effect of the Treatments on Cell Death Induction (Apoptosis and Autophagy)

**Figure 7:**
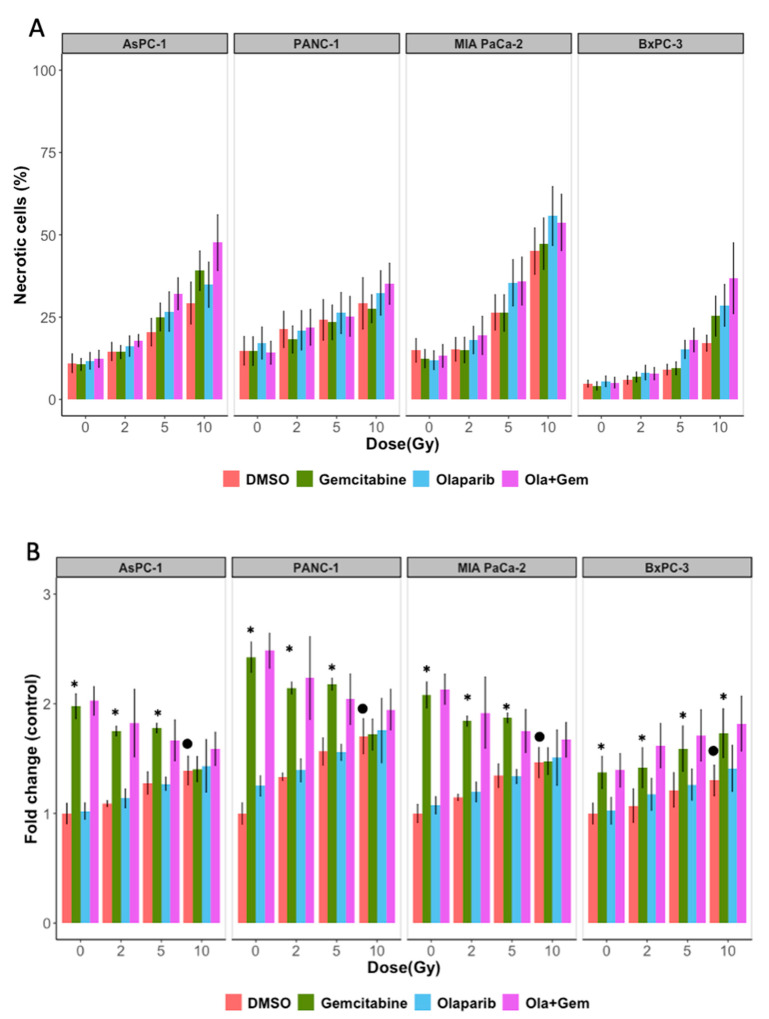
(**A**) Effect of irradiation (2, 5 and 10 Gy), olaparib (1 µM) and gemcitabine (10 nM) on PANC-1, AsPC-1, MIA PaCa-2 and BxPC-3 necrosis cell death quantifications. Cells were labelled 48 h after treatment with Annexin V and propidium iodide (PI). Cells marked as Annexin V-/PI+ were determined by flow cytometry for necrosis. (**B**) Effect of irradiation (2, 5 and 10 Gy), olaparib (1 µM) and gemcitabine (10 nM) on PANC-1, AsPC-1, MIA PaCa-2 and BxPC-3 autophagic induction. Cells were labelled 24 h after treatment using Cyto-ID^®^ Green detection reagent according to protocol described in materials and methods, and the mean fluorescence was determined by flow cytometry. Statistical significance is indicated: * *p* < 0.05 (vs. control/irradiation). • *p* < 0.05 (vs. Control).

Next, we determined what type of cell death was induced by treatment with olaparib, gemcitabine and irradiation, individually or in combination on pancreatic cancer cell lines. Percentages of cells undergoing apoptotic cell death were determined at 24 and 48 h after treatment. We showed that apoptosis levels were very low at 24 and 48 h in all groups of treated cells, independently of the radiation dose (data not shown). In contrary, necrosis was enhanced in dose dependent manner for all cell lines. We then evaluated necrosis after 10 Gy irradiation, and showed that necrosis induction was dependent on the type of cell line and the type of drug associated with irradiation (Figure 7A and Table 4) but the results were not significant.

We then assessed autophagy as other mechanism of cell death. The results showed that both high dose irradiation (10 Gy) and gemcitabine treatments alone induced autophagy whereas treatment with olaparib did not induce autophagic cells compared to non-treated cells. When cells were co-treated with gemcitabine and olaparib, autophagy induction was not significantly higher than in gemcitabine treated cells alone (Figure 7B).

## 3. Discussion

Effective treatments for locally advanced pancreatic cancer are limited. Therefore, any new modality to replace or support current treatments for pancreatic cancer would be highly valuable.

In this study, we evaluated in vitro efficacy of the association of gemcitabine, olaparib and irradiation in four locally advanced pancreatic cancer cell lines. First, our results indicated that this triple association reduced cell growth and clonogenic survival, compared to both gemcitabine/irradiation or olaparib/irradiation combinations and standard treatment combining chemoradiotherapy with gemcitabine. This effect was mainly due to the radiosensitizing effect of olaparib. Indeed, we depicted that although olaparib alone at a concentration of 1 µM was not toxic for all four cell lines as chosen for this project, it had a clear radiosensitizing effect and particularly with high dose of radiation (10 Gy). This is consistent with results obtained by Vance et al., who treated MIA PaCa-2 cells with 1 µM of olaparib and increasing doses of irradiation (ranged from 2 to 8 Gy) and found a radiation enhancement ratio of 1.5 ± 0.1, whereas cytotoxicity of olaparib alone was 1.1 ± 0.1 [5]. It is also coherent with Karnak et al. who reported radiosensitization of AsPC-1 cell line with olaparib (SER = 1.2 ± 0.2) [6]. We also studied two other pancreatic cell lines that have never been studied before with olaparib, PANC-1 and BxPC-3. We pointed out that the magnitude radiosensitization with olaparib was dependent of the cell line. As cell lines have different genomic background, the extent of radiosensitization could be due to their genomic alterations. Indeed, our data showed that olaparib and gemcitabine have a synergistic radiosensitization effect on BxPC-3 cell line but not in three other cell lines. The combination of olaparib with gemcitabine-based chemoradiotherapy should then be assessed in vivo.

Another aspect of the present study was the investigation of cell cycle perturbation with PARP inhibitors. We and others have shown that PARP-1 inhibition resulted in a greater accumulation of cells in the G2/M phase in response to radiation, likely due to persistent DNA damage, whereas gemcitabine blocked cells in early S-phase, likely due to the inhibition of replication [7,8,9]. As G2 checkpoint activation is known to be a consequence of persistent DNA damage, we assessed DSBs repair through residual γ-H2Ax foci, 24 h after treatments and showed that olaparib combined with irradiations (10 Gy) increased γ-H2AX foci compared to control irradiated cells. To properly compare the radiosensitization effect of gemcitabine with olaparib, we assessed radiosensitization effect of a non-cytotoxic concentration of gemcitabine (10 nM). As Pauwels et al. presented in different types of cancer cell lines, radiosensitization with gemcitabine was time-exposure dependent and was higher with 24 h exposure before irradiation [10], therefore we treated cell lines with 24 h of gemcitabine before irradiation. To display radiosensitization with CHK1/2 (AZD7762) inhibitor in pancreatic cancer, Morgan et al. irradiated and co-treated MIA PaCa-2 cells with gemcitabine/AZD7762 and showed a radiation enhancement ratio of 1.5 with gemcitabine treatment alone. In their treatment schedules, cells were treated 24 h before irradiation with gemcitabine and at time of irradiation, approximately 45% of cells were in S-phase [11]. Indeed, Im et al. presented that radiosensitization with gemcitabine required a depletion in deoxynucleotide for approximately 4 h with accumulation of cells in S-phase [12]. These observations are coherent with our hypothesis that gemcitabine could synchronize cell lines into S-phase, thus leading to enhanced sensitization after irradiation and olaparib treatment. As S-phase is known to be a radioresistant phase, olaparib treatment could overcome intrinsic radioresistance.

Ewald et al. showed that H2AX phosphorylation was not only associated with DSBs but also with agents that inhibits DNA synthesis, such as gemcitabine and was due to stalled replication forks during S-phase [13]. Moreover, they displayed that some γ-H2AX foci co-localized with DNA damage response proteins (Mre11, Rad50 and Nbs1) at the site of stalled replication forks, shortly after nucleoside analogue exposure. More recently, Bryant et al. presented that treatment with hydroxyurea induced stalled replication forks, thus stimulating PARP-1 activation, leading to recruitment of Mre11 and Nbs1, thus promoting repair through homologous recombination [14]. Thus, there is an interplay between gemcitabine inducing stalled replication forks, PARP activation and homologous recombination. The radiosensitizing effect of PARPi may be due to different mechanisms. First, PARP inhibitor inhibits enzymatic activity of PARP, thus decreasing protein PARylation. Besides, PARPi efficacy may also be attributed to PARP trapping, whereby it remains to be restrained at the site of DNA damage, hence preventing DNA repair. Recently, Parsels et al. showed that PARP trapping with olaparib and ATR inhibition could overcome intrinsic resistance of HR-proficient pancreatic cancer cells [15].

We also reported that cell death was partly induced by autophagy, specifically for gemcitabine-base treatments, but apoptosis was clearly not involved. Our results are consistent with the literature. Indeed, Chen et al. showed that even with the highest concentration of olaparib (1 µM), there was at most 6% of apoptosis in PANC-1 and BxPC-3 pancreatic cancer cell lines [16]. More recently, Pandita et al. displayed that in MIA PaCa-2 cell line, treatment with 660 nM of gemcitabine resulted in 2% of apoptosis [17]. MIA PaCa-2 cells seemed to be more resistant to gemcitabine. This was partly explained by the fact that gemcitabine induced apoptosis through p53 pathway and mutant p53 cells [18]. Autophagy is a conserved process by which cytoplasm and cellular organelles are degraded in lysosomes. There is much controversy concerning the role of treatment induced-autophagy in pancreatic cancer. Authors argued that gemcitabine-induced autophagy prevents pancreatic carcinoma entering in apoptotic pathway, thus allowing resistance to treatment [19,20]. Others claimed that autophagy constituted a death mechanism, when pancreatic cancer cells were treated with gemcitabine and/or ionizing radiation [18,20]. Indeed, Rosenfeldt et al. used a humanized genetically modified mouse model of PDAC and showed that autophagy’s role in tumor development was connected to the status of p53. Indeed, loss of autophagy in tumor lacking p53 accelerated tumor onset, whereas it blocked progression to high grade malignant tumors in p53 wild-type cells [20]. These data are consistent with results published by Fiorini et al., showing in a mutant p53 cell line, that incubation of gemcitabine with p53 reactivating molecules, induced autophagy and apoptosis. However, inhibiting autophagy enhanced anti-proliferative activity of combined treatment, thus demonstrating that autophagy induced by gemcitabine in p53 wild-type cell line may have a pro-survival effect [18]. Here, we presented on four p53 mutant cell lines, that gemcitabine induced autophagy compared to control, but this effect was clearly attenuated with increasing radiation doses, and did not seem to be synergic. On the other hand, surviving fraction and cell growth were decreased with irradiation and gemcitabine compared to control, and the addition of olaparib also decreased surviving fraction. These data suggest that some other types of cell death contribute to the effectiveness of the combined triple treatment with gemcitabine, olaparib and irradiation. Several studies have examined the mechanism of cell-death induced by PARP inhibition. Some authors explored senescence as a mode of cell death after irradiation and treatment with PARPi. A radiosensitization of tumor cells was observed via the promotion of senescence [21]. Another induced cell death could be mitotic catastrophe [8].

Given the lack of effective strategies to treat pancreatic cancer and based on these data showing that PARPi could radiosensitize PDAC cell lines, targeting DNA damage through PARP inhibition should be explored as therapeutic options to treat locally advanced PDAC.

## 4. Materials and Methods

### 4.1. Cell Culture

The human pancreatic cancer cell lines MIA PaCa-2, AsPC-1, PANC-1 and BxPC-3 were purchased from American Type Culture Collection (CRL-1420TM, CRL-1682, CRL-1469 and CRL-1687, respectively, ATCC; Rockville, MD, USA) and banked at Centre Paul Strauss. Cells were cultured at 37 °C in a humidified atmosphere containing 5% CO_2_ and 95% air. PANC-1 was cultured in Dulbecco’s modified Eagle’s medium (DMEM; PAN Biotech GmbH, Aidenbach, Germany) supplemented with 10% fetal bovine serum (FBS, PAN Biotech GmbH) and 1% of a solution of penicillin (10000 IU/mL) and streptomycin (10 mg/mL) (PAN Biotech GmbH). MIA PaCa-2 was cultured in the same conditions with 1% of 4-(2-hydroxyethyl)-1-piperazineethanesulfonic acid (HEPES, 1 mM, PAN Biotech GmbH), 1% of sodium pyruvate (10 mM, PAN Biotech GmbH) and 1% of non-essential amino acids (NEAA, PAN Biotech GmbH). AsPC-1 and BxPC-3 were cultured in Roswell Park Memorial Institute medium (RPMI; PAN Biotech GmbH) supplemented with 10% FBS, and 1% of a solution of penicillin (10000 IU/mL) and streptomycin (10 mg/mL). Subconfluent cell monolayers were trypsinized once a week using 0.5% trypsin containing 2% EDTA (PAN Biotech GmbH) and plated at passage ratios between 0.25:10 to 1:10, according to the cell line or used directly for study after enumeration determined with a Countess^®^ Cell Counter (Countess, Invitrogen, Carlsbad, CA, USA).

### 4.2. Drugs and Chemicals

Olaparib and gemcitabine were provided by Selleck Chemicals (Houston, TX, USA). Stock solutions were prepared at 10 mM in DMSO and stored in aliquots at −20 °C. Olaparib was used at 1 µM and gemcitabine at 10 nM.

### 4.3. Irradiation Exposure

Cells seeded in 6-well plates were exposed, at room temperature, to photon irradiation, one hour after pharmacological treatment (olaparib and/or gemcitabine), at one fraction-doses of 2, 5 and 10 Gy. A ^137^Cs γ-irradiator (Biobeam GM 8000, GSM GmbH, Leipzig, Germany) was used in the Paul Strauss Center (Strasbourg, France) at a dose rate of 2.5 Gy/min. Control flasks were sham irradiated.

### 4.4. Clonogenic Survival Assay

Cells were seeded in 6–well plates at a density of 2 × 10^5^ cells per well and allowed to adhere overnight in standard culture conditions. Cells were exposed to DMSO (control), olaparib and/or gemcitabine 1 h before irradiation with 2, 5 or 10 Gy. Following treatment for 24 h, cells were trypsinized, collected and numbered. Then, cells were seeded at optimized densities according to radiation dose and plated at two different dilutions into 6-well plates. Twelve to nineteen days later, depending on cell lines, the clones were stained using 0.05% crystal violet (Sigma-Aldrich, Lyon, France) in 5% ethanol solution and positive colonies (≥50 cells) were scored. The plating efficiency (PE) and then the surviving fractions were calculated. Survival curves were plotted using surviving fractions for different doses. To generate the radiation dose–response curves, the data was fitted to the linear quadratic (LQ) model, where S(D) is the fraction of cells surviving a dose of D and α/β are inactivation constants: S(D) = e^(−αD−βD^2). Semi-logarithmic survival curves were constructed for determination of survival ability of cells in response to various treatments. The linear-quadratic (LQ) cell survival curve parameters were calculated with CFAssay Package using R Software. SER were determined by calculating the ratio of doses in treated and control conditions for a given isoeffect (SF = 0.1).

### 4.5. PARP Inhibitor Activity

Cells were pre-incubated with olaparib (1 µM) during 1 h. The treated cells were then exposed to 500 µM of H_2_O_2_ for 10 min. Cells were fixed with a solution of methanol/acetone 1:1 at 4 °C for 20 min. Next, cells were permeabilized by three washes with PBS-tween. The poly(ADP-ribose) (PAR) signal was then detected by incubation with the anti-PAR antibody (1/1000; clone 10H Sugimura) in PBS-tween and BSA 0.1% for 2 h at 4 °C. Then, the cells were washed three times with PBS-tween. The cells were incubated with the secondary mouse antibody anti-IgG coupled with Alexa 594 (1/2000; Invitrogen) in PBS-tween and BSA 0.1% for 2 h at 4 °C. Subsequently, cells were washed with PBS-tween, next PBS and finally PBS-DAPI. Last, cell glass slides were prepared by fixation with Mowiol and antifading agent DABCO. Finally, detection of PAR was analyzed by immunofluorescence microscopic imaging using an Olympus BH-2 fluorescent microscope equipped with a digital camera.

### 4.6. Cell Cycle Distribution

Twenty-four hours after treatments, treated cells were trypsinized, harvested and washed twice in phosphate buffered saline (PBS, pH 7.2) fixed in 1 mL of ice-cold 70% ethanol and stored at −20 °C. The pellet was resuspended in 200 μL PBS containing 1 mg/mL RNase A, and the cellular DNA was stained with propidium iodide (PI; 0.1 mg/mL; Sigma, Lyon, France). Cells were incubated at 37 °C in the dark for 30 min prior to analysis. Cell cycle determination was performed using a BD Accuri^TM^ C6 flow cytometer (Becton Dickinson, BD, San Jose, CA, USA) and fluorescence of at least 10,000 cells were analyzed using BD Accurri and the ModFit softwares, provided by the manufacturer.

### 4.7. Apoptotic and Necrotic Detection Assay

Apoptotic cells were quantified 24 and 48 h after irradiation. Harvested cells were washed with PBS and resuspended in 200 μL of 1X Annexin V Binding Buffer (FITC Annexin V Apoptosis Detection kit, BD Pharmingen). Then, 5 μL of Annexin V and 5 μL of propidium iodide (PI) were added to 100 μL of the solution. After incubation in the dark at room temperature for 15 min, 200 μL 1X Annexin V Binding Buffer was added to the solution and the fluorescence of 10,000 cells was analyzed using a BD Accuri^TM^ C6 flow cytometer and software (BD). Annexin V+/PI− cells were recorded as being early apoptotic, whereas Annexin V+/PI+ were considered to be necrotic.

### 4.8. Autophagic Detection Assay

For autophagy determination 24 h after irradiation, we used the Cyto-ID^TM^ Autophagy Detection kit (Enzo Life Sciences, Plymouth Meeting, PA, USA) according to the manufacturer’s instructions. Cells were washed in PBS (pH 7.2) and resuspended in 500 μL PBS containing Cyto-ID^®^ Green Detection Reagent (0.1% *v*/*v*). Then, cells were incubated at 37 °C in the dark for 30 min and were resuspended in 200 μL PBS. The fluorescence emission of 10,000 cells was analyzed using a BD Accuri^TM^ C6 cytometer and software (BD).

### 4.9. Determination of γ-H2AX Formation

For γ-H2AX foci visualization, cells grown on Lab-Tek chambers (Nunc, Thermofisher, Illkirch, France) were treated as indicated earlier and fixed with a 4% paraformaldehyde solution for 15 min. Then samples were permeabilized with PBS containing 0.5% Triton-X-100 and blocked with 10% BSA. Then, cells were incubated overnight at 4 °C, with blocking buffer containing primary antibody against γ-H2AX (clone JBW301; Merck Millipore, Darmstadt, Germany) followed by incubation with Alexa Fluor 488 goat anti-mouse IgG (Invitrogen) for 3 h at room temperature. Samples were counterstained with antifading agent containing DAPI. The formation of foci in nuclei were monitored by immunofluorescence using an Olympus BH-2 fluorescence microscope equipped with digital camera.

For γ-H2AX analysis in flow cytometry, 24 h after treatment, cells were harvested and washed in ice-cold PBS, fixed in 1 mL of 70% ethanol and stored at −20 °C for 24 h. Fixed cells were centrifuged and pellet was resuspended twice in 2 mL cold PBS containing 10% bovine serum albumin (BSA, Sigma-Aldrich, Lyon, France) and 0,2% Triton-X-100 (T-PBS-BSA). Then, labelling was performed using a solution of monoclonal mouse anti-phospho-histone-H2AX (ser 139) polyclonal antibody (clone JBW301; Merck Millipore, Darmstadt, Germany) diluted in 1/100 in T-PBS-BSA. Cells were stored at 4 °C overnight. Cells were washed twice with 2 mL T-PBS-BSA and resuspended in 100 µL of a solution containing an anti-mouse IgG fluorescein-conjugated antibody diluted at 1:200 (Invitrogen) and were stored 1 h in room temperature and gently shaken. Then, 2 mL T-PBS-BSA wad added, cells were centrifuged and pellet was resuspended in 200 µL of PBS containing PI (5 µg/mL) and RNAse A (100 µg/mL). Next, cells were stored at room temperature in the dark for 30 min and gently shaken. The fluorescence of 10,000 cells was analyzed using a BD Accuri^TM^ C6 flow cytometer and software (BD). For quantification of γ-H2AX positivity, a gate was arbitrarily set on the control, untreated sample to define a region of positive staining for γ-H2AX of approximately 5% [22]. This gate was then overlaid on the treated samples.

### 4.10. Statistical Analysis

Experiments were performed at a minimum in triplicate and results were expressed as the mean ± standard deviation (SD) unless otherwise indicated. Statistical differences of data were assessed by the *t*-test using R Software (ver. 3.4.0 http://cran.r-project.org/). *p*-Values lower than 0.05 were considered as statistically significant. For clonogenic survival assay, results from 4 experiments were subjected to linear-quadratic regression analysis, using the maximum likelihood approach. Differences between curves were evaluated using a two-way ANOVA test.

## 5. Conclusions

Generally, our results showed that PDAC cell lines could be sensitized by irradiation and olaparib treatments, through an increase of unrepaired DSBs and a block in G2 phase. Moreover, we displayed that this radiosensitizing effect was greater with higher dose per fraction.

Our work could have clinical impact for patients with locally advanced PDAC. Indeed, the low response rate after chemoradiation (52%) could be enhanced by the radiosensitization effect of olaparib [23]. Moreover, as olaparib radiosensitized cells when treated with high dose of radiation, patients could benefit from advanced modality treatment technology such as stereotactic radiotherapy, which can deliver high dose per fraction in a highly conformal way. Finally, we need to investigate effect of gemcitabine, olaparib and irradiation on an in vivo model of PDAC.

## Figures and Tables

**Table 1:** Surviving fraction calculated after treatment with olaparib (1 µM) or gemcitabine (10 nM) for AsPC-1, BxPC-3, MIA PaCa-2 and PANC-1 cell lines.

Treatment	AsPC-1	BxPC-3	MIA PaCa-2	PANC-1
Olaparib	1.05 ± 0.06	0.93 ± 0.05	1.02 ± 0.03	0.97 ± 0.02
Gemcitabine	0.98 ± 0.03	0.96 ± 0.03	1.01 ± 0.02	0.95 ± 0.02

**Table 2:** Values of linear quadratic parameters (α and ß) and standard enhancement ratio (SER) from PDAC cells treated with ionizing radiation and olaparib and/or gemcitabine. Statistical significance is indicated: * *p* < 0.05 (vs. control). • *p* < 0.05 (vs. Gemcitabine).

Cell Line	Treatment	α (Gy-1)	ß (Gy-2)	SER
MIA PaCa-2	DMSO	0.454	0.025	1.0
	Gemcitabine	0.404	0.040	1.04 ± 0.01
	Olaparib *	0.515	0.048	1.24 ± 0.001
	Olaparib + Gemcitabine *^,^•	0.590	0.041	1.30 ± 0.02
	Expected combined SER			(1.29 ± 0.02)
PANC-1	DMSO	0.273	0.024	1.0
	Gemcitabine	0.250	0.032	1.04 ± 0.01
	Olaparib *	0.402	0.041	1.38 ± 0.01
	Olaparib + Gemcitabine *^,^•	0.291	0.077	1.48 ± 0.05
	Expected combined SER			(1.45 ± 0.001)
AsPC-1	DMSO	0.438	0.033	1.0
	Gemcitabine	0.520	0.053	1.22 ± 0.01
	Olaparib *	0.319	0.098	1.21 ± 0.06
	Olaparib + Gemcitabine *^,^•	0.367	0.138	1.45 ± 0.08
	Expected combined SER			(1.47 ± 0.09)
BxPC-3	DMSO	0.267	0.013	1.0
	Gemcitabine	0.640	−0.006	1.75 ± 0.01
	Olaparib *	0.374	0.004	1.02 ± 0.02
	Olaparib + Gemcitabine *^,^•	0.779	−0.015	2.07 ± 0.07
	Expected combined SER			(1.79 ± 0.05)

**Table 3:** Percentages of cell in S phase after control (CTL) and gemcitabine (10 nM) for AsPC-1, BxPC-3, MIA PaCa-2 and PANC-1cell lines. * *p* < 0.05 (vs. control).

Cell line	CTL	Gemcitabine
AsPC-1	22.29 ± 2.23	28.17 ± 0.80
MIA PaCa-2	22.73 ± 1.29	32.50 ± 3.59 *
PANC-1	22.26 ± 1.69	25.03 ± 1.56
BxPC-3	21.23 ± 2.23	26.83 ± 0.77

**Table 4:** Mean values of necrotic cellsfrom PDAC cell lines at 48 h after 10 Gy irradiation.

Cell Line	Treatment	Necrotic Cells (%) at 10 Gy
MIA PaCa-2	DMSO	45.1
	Gemcitabine	47.3
	Olaparib	55.7
	Olaparib + Gemcitabine	53.8
PANC-1	DMSO	17.1
	Gemcitabine	25.3
	Olaparib	28.6
	Olaparib + Gemcitabine	36.8
AsPC-1	DMSO	29.3
	Gemcitabine	39.1
	Olaparib	34.9
	Olaparib + Gemcitabine	47.6
BxPC-3	DMSO	29.2
	Gemcitabine	27.6
	Olaparib	32.4
	Olaparib + Gemcitabine	35.2

## Data Availability

Not applicable.
